# Ribozyme inhibition of the protein kinase C**α** triggers apoptosis in glioma cells

**DOI:** 10.1038/sj.bjc.6690560

**Published:** 1999-07

**Authors:** M Leirdal, M Sioud

**Affiliations:** Institute for Cancer Research, Department of Immunology, The Norwegian Radium Hospital, N-0310 Oslo, Norway

**Keywords:** ribozyme, gliomas, protein kinase C, apoptosis

## Abstract

Although protein kinase C has been shown to be involved in a wide range of biological functions, the precise role of each isoform in a specific cell function remains to be clarified. Here we demonstrate that a ribozyme specific for the human protein kinase Cα (PKCα), a classical PKC isoform, induces cell death in glioma cell lines. This cell death was identified as apoptosis by morphologic alterations and endonucleosomal DNA fragmentation. The inhibition of PKCα gene expression by the ribozyme resulted in a significant reduction in Bcl-x_L_ gene expression, a protein that inhibits apoptosis and is overexpressed in glioma cells. Taken together, our data suggest that the PKCα ribozymes are a potent inducer of apoptosis in glioma cells, which may act through suppressing Bcl-x_L_ gene expression and/or activity. PKCα ribozymes may prove useful in the management of malignant gliomas. © 1999 Cancer Research Campaign


*British Journal of Cancer* (1999) **80**(10), 1558–1564
© 1999 Cancer Research Campaign
Article no. bjoc.1999.0560

### Ribozyme inhibition of the protein kinase Cα triggers apoptosis in glioma cells

**M Leirdal and M Sioud**

Institute for Cancer Research, Department of Immunology, The Norwegian Radium Hospital, N-0310 Oslo, Norway

**Summary** Although protein kinase C has been shown to be involved in a wide range of biological functions, the precise role of each isoform in a specific cell function remains to be clarified. Here we demonstrate that a ribozyme specific for the human protein kinase Cα (PKCα), a classical PKC isoform, induces cell death in glioma cell lines. This cell death was identified as apoptosis by morphologic alterations and endonucleosomal DNA fragmentation. The inhibition of PKCα gene expression by the ribozyme resulted in a significant reduction in Bcl-$x_L$ gene expression, a protein that inhibits apoptosis and is overexpressed in glioma cells. Taken together, our data suggest that the PKCα ribozymes are a potent inducer of apoptosis in glioma cells, which may act through suppressing Bcl-$x_L$ gene expression and/or activity. PKCα ribozymes may prove useful in the management of malignant gliomas.

**Keywords:** ribozyme; gliomas; protein kinase C; apoptosis

Apoptosis, programmed cell death, is a process of active cellular self-destruction that plays a crucial role in cell development and homeostasis. In mammalian cells this process is regulated by a number of gene products. Among them, Bcl-2, Bcl-$x_L$ and Mcl-1 inhibit apoptosis, whereas others, such as Bax, Bak and Bad, activate apoptosis (Kroemer, 1997). Dysregulation of genes involved in apoptosis can contribute to drug resistance. For example, overexpression of the Bcl-2 protein has been shown to block apoptosis that is induced by a number of different stimuli, including anticancer drugs (Pegoraro et al, 1984; Rodin and Thompson, 1997). Thus, analysis of the signal pathways that regulate the expression and/or the activity of the Bcl-2 related proteins (e.g. Bcl-$x_L$ and Mcl-1) may lead to the development of a new treatment based on the induction of apoptosis in tumour cells.

Protein kinase C (PKC) is a multigene family of serine/threonine kinases that are central to many signalling pathways regulating cell growth and differentiation (Nishizuka, 1992; Dekker and Parker, 1994). Recent studies have shown that PKC inhibitors block cancer cell growth via induction of apoptosis (Couldwell et al, 1994; Ikemoto et al, 1995; Qiao et al, 1996). Notably, glioma cells contain a high PKC activity when compared to non-transformed glial cells (Couldwell et al, 1991). This increased PKC activity in glioma cells has been used as a target for inhibition by PKC inhibitors (Couldwell et al, 1994; Ikemoto et al, 1995). However, the role of each PKC isoforms in the sustained glioma cell growth was not established, since the used drugs do not discriminate between the 12 characterized PKC isoforms (Dekker and Parker, 1994). Furthermore, in most studies the potential signals that link PKC to apoptosis were not identified, and some of the currently used PKC inhibitors were also found to block the protein tyrosine kinase activity (Meggio et al, 1995). Accordingly, we have focused our attention on examining the molecular mechanisms by which PKC isoform-specific inhibitors inhibit glioma cell proliferation. Such specific agents might be useful as pharmaceuticals.

Because of the high specificity of Watson–Crick base pairing, in principle strategies based upon nucleic acids targeted at a gene coding for a pathogenic protein should interfere only with its function. For therapeutic application, cleavage of messenger RNAs by catalytic RNAs (ribozymes) is likely to be more promising than, for example, the antisense and antibody approaches. Ribozymes hybridize specifically to complementary mRNA and block the encoded protein by cleaving its mRNA (Haseloff and Gerlach, 1988; Cameron and Jennings, 1989; Cotton and Birnstiel, 1989; Sarver et al, 1990; Sioud and Drlica, 1991; Sioud et al, 1992; Scanlon et al, 1994; Sioud, 1996). By introducing 2′-amino pyrimidine residues into ribozymes, recently we have developed a nuclease resistant PKCα ribozyme that blocked glioma tumour growth in inbred syngeneic rats (Sioud and Sørensen, 1998). Other investigators have demonstrated that antisense against PKCα can block, for example, human lung carcinoma growth in nude mice (Dean et al, 1996), suggesting a crucial role of PKCα in various tumour growth. In searching for potential mechanisms that might explain the inhibition of glioma cell proliferation by ribozymes, in the present study we demonstrate that inhibition of PKCα leads to a decrease in Bcl-$x_L$ gene expression and consequent induction of apoptosis.

*Received 22 October 1998*
*Revised 2 February 1999*
*Accepted 9 February 1999*

*Correspondence to:* M Sioud

### MATERIALS AND METHODS

### Cell lines

Human T98G and U87MG glioblastoma cell lines were obtained from American Tissue Type Culture (ATCC), and grown in Dulbecco’s modified Eagles medium (DMEM) supplemented with 10% fetal bovine serum (FBS) according to the instructions of ATCC.

### Western analysis

Cytoplasmic extracts were prepared from control and test molecule-treated cells according to reference (Sioud, 1994; Sioud and Jespersen, 1996). Extracts (15 μg per lane) were separated by electrophoresis on a 10% polyacrylamide gel under denaturing conditions. Proteins were transferred to nitrocellulose membrane and immunoblotted with a rabbit IgG polyclonal anti-PKCα, anti-PKCβII, antiPKCα, anti-PKCδ, anti-PKCEε, anti-PKCE, anti-Bcl-$x_L$ or anti-Bax antibodies (Santa Cruz Biotechnology), and visualized by the ECL-system (Amersham) using horseradish peroxidase conjugated anti-rabbit IgG (Sigma).

### Subcellular fractionation

Cells were washed in ice-cold phosphate-buffered saline (PBS) and resuspended in buffer A (5 mM Tris–HCl, pH 8, 0.5 mM EDTA, 75 mM sucrose and proteinase inhibitors) and then sonicated four times, 15 s each. Complete cell lysis was confirmed by microscopy. Nuclei were pelleted by centrifugation at 2000 rpm for 5 min at 4°C in a microcentrifuge. The supernatants were centrifuged at 40 000 rpm for 30 min at 4°C in a Beckman ultracentrifuge. Each supernatant was collected and used as the cytosol fraction. The membrane pellets were washed three times with PBS, solubilized in buffer A containing 1% Triton X-100 for 15 min at 4°C and then centrifuged at 15 000 rpm for 10 min at 4°C in a microcentrifuge. Supernatants were used as the membrane fraction. In all cases, protein concentrations were determined using the protein assay kit (BioRad).

### MTT (tetrazolium) assay

Cells were resuspended in DMEM containing 10% FBS at a concentration of $2 \times 10^4$ cells $ml^{-1}$ and 100 μl aliquots ($2 \times 10^3$ cells) were plated into 96-well, flat-bottom tissue culture plates. The plates were incubated at 37°C for at least 6 h to allow recovery of the cells from trypsinization. Following incubation, cells were transfected with the test molecules in complete medium using DOTAP as described previously (Sioud, 1994). After 48 h transfection time, stock MTT (3-4,5-dimethylthiazol-2-yl)-2-5-diphenyltetrazolium bromide) solution (10 μl per 100 μl medium) was added to all wells, and the plates were incubated at 37°C for 4 h. Acid-isopropanol (100 μl of 0.04 N hydrochloric acid in isopropanol) was added to all wells and mixed thoroughly to dissolve the formed crystals, the plates were read using a test wavelength of 570 nm and a reference wavelength of 630 nm.

### In vitro RNA synthesis

An asymmetric 2′-amino modified ribozyme having as a cleavage site the GUU corresponding to the codon number 4 within the human PKCα mRNA were synthesized by in vitro transcription using DNA oligodeoxynucleotide and the T7 RNA polymerase as described previously (Sioud and Drlica, 1991). In brief, to generate the ribozyme minigene, two overlapping half deoxynucleotides containing the T7 promoter sequence and the sequence coding for the catalytic centre and the flanking regions of each ribozyme were annealed and then extended with the Klenow enzyme. After extension, the DNA was polyacrylamide gel purified and then used as template for in vitro transcription. Following transcription RNA was gel purified. The ribozyme sequence is: 5′ GGGAACUGAUGAGUCCGUGAGGACgAAACGUCAGCCA-UGG 3′. Ribozyme with only antisense activity, mutant ribozyme, was made by deleting the G12 from the catalytic core as indicated by lower case letter (for numbering see Hertel et al, 1992).

### Total RNA preparation and RT-PCR

Total RNA was prepared from control and test molecule-treated cells according to Chomczynski and Sacchi (1987) and 1 μg was reverse transcribed (RT) using the first strand cDNA synthesis kit and oligo dT primers as recommended by the manufacturer (Pharmacia, Uppsala, Sweden). Polymerase chain reaction (PCR) was performed on entire cDNA product by using *Taq* DNA in a gene Amp PCR system 2400 (Perkin-Elmer/Cetus) using primers specific for the PKCα isoform. Following 30 cycles of amplification, PCR products were separated in a 1.5% agarose gel and visualized by staining with ethidium bromide. As a control, actin was co-amplified using specific primers.

### TUNEL-reaction

A commercially available in situ cell death fluorescein detection kit based upon terminal deoxynucleotidyl transferase (TdT)-mediated dUTP-FITC nick end labelling (TUNEL) was used (Boehringer). Briefly, cells were washed with PBS and fixed in 4% paraformaldehyde solution in PBS. Then after washing with PBS cells were permeabilized with 0.1% Triton X-100 in 0.1% sodium citrate for 2 min at 4°C. Following washing with PBS, cells were incubated with the TUNEL reaction for 30 min, washed with PBS and then analysed by flow cytometry.

### Detection of DNA fragmentation following ribozyme treatment

After ribozyme treatment, cell pellets were lysed in 0.02% N-lauryl-sarcosine (Sigma) in 50 μl TE buffer. Ribonuclease A was added and lysates were incubated at 37°C for 30 min. Thereafter, proteinase K was added and samples were incubated at 37°C for another 60 min. Following incubation, the resultant crude DNA preparations were analysed by electrophoresis on a 1% agarose gel and visualized by ethidium bromide staining.

### Statistical analysis

Each experiment was performed at least four times. Statistical significance of ribozyme and mutant ribozyme effects on cell proliferation, PKC and Bcl-$x_L$ gene expression were assessed by unpaired Student's *t*-test.

### RESULTS

### Human glioma cell lines upregulate the expression of PKCα and Bcl-$x_L$ proteins

Recently we have found that rat glioma cell lines overexpress the PKCα isoform and the anti-apoptotic protein Bcl-$x_L$, compared with, for example, the expression of the PKCδ isoform and Bcl-2 protein (Sioud and Sørensen, 1998). To determine whether the upregulation of the PKCα and the Bcl-$x_L$ gene expression is also a property of human glioma cells, we performed experiments with human glioma cell lines. As illustrated in Figure 1A, the T98G and

© 1999 Cancer Research Campaign

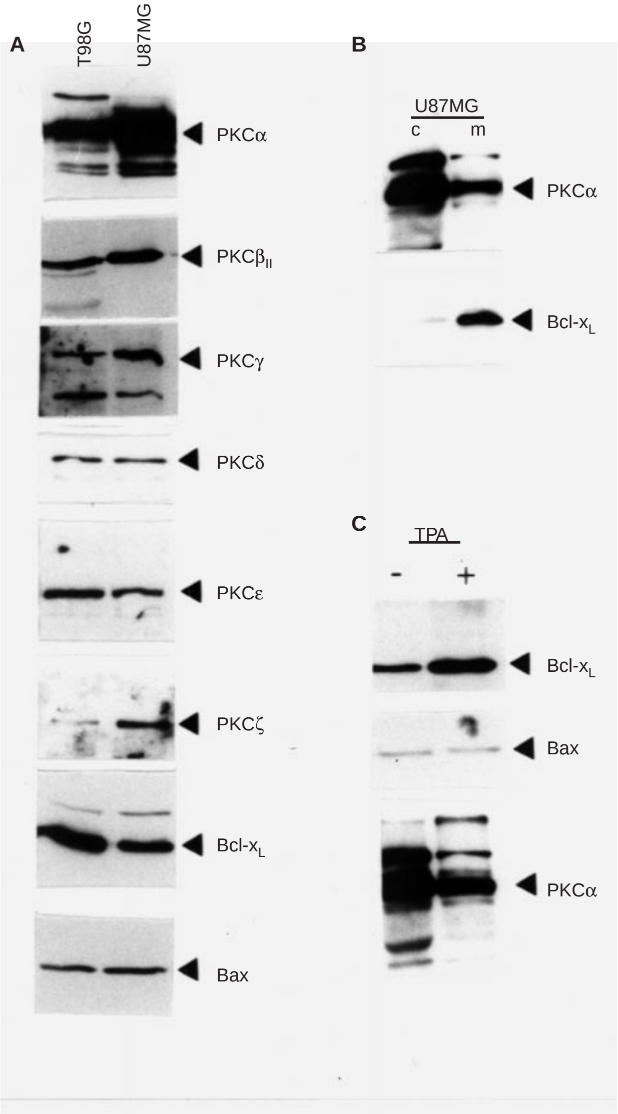

**Figure 1** Western blot analysis. (**A**) Expression of the PKC isoforms, the Bcl-$x_L$, and the Bax proteins by T98G and U87MG human glioblastoma cell lines. Both glioma cell lines overexpress the PKCα and Bcl-$x_L$ proteins. (**B**) Analysis of PKCα and Bcl-$x_L$ in the cytosol and the membrane fractions prepared from U87MG glioma cells. m = membrane fraction, c = cytosol fraction. Similar results were obtained with T98G cells. (**C**) Up-regulation of Bcl-$x_L$ gene expression by TPA. Cells were incubated with TPA for 5 h. Protein extracts from unstimulated and stimulated cells were prepared and 15 μg from each sample were analysed by immunoblotting using Bcl-$x_L$, Bax or PKCα antibodies. Bands that represent the investigated proteins are indicate by arrows. Data are representative of four independent experiments

U87MG cell lines overexpress the PKCα and the Bcl-$x_L$ proteins. The expression of Bcl-2 protein by both cell lines was very weak and in many cases undetectable (data not shown). A significant fraction (15%) of the PKCα was found to be associated with membrane fraction (Figure 1B). As expected, the Bcl-$x_L$ is mainly a membrane-bound protein.

Because many gene products have been shown to be under the control of the PKC signal pathways (Nishizuka, 1992; Newton, 1995), therefore we investigate whether the activation of PKC by phorbol esters (e.g. TPA) would increase Bcl-$x_L$ gene expression in glioma cells. In principle, binding of TPA to the amino-terminal regulatory regions of some PKC isoforms, in particular PKCα, would induce conformational changes, resulting in their activation, membrane translocation and sensitivity to proteolytic cleavage (Nishizuka, 1992). The time course for PKC activation, depletion and their de novo synthesis following TPA stimulation varies significantly with cell types (Parker et al, 1989; Mischak et al, 1993). Exposure of U87MG cells to TPA for 5 h led to a threefold increase in Bcl-$x_L$ gene expression, while the Bax gene expression was not affected by this treatment (Figure 1C). The level of PKCα decreased in this TPA-treated cells. Long-term TPA (100 nM) stimulation of glioma cells induced PKCα down-regulation, but not depletion (data not shown), suggesting an active de novo synthesis.

### Effect of the PKCα ribozyme on cell proliferation and on PKCα gene expression

To evaluate the effect of the PKCα isoform on the human glioma cell proliferation, we have targeted its expression by a ribozyme. As shown in Figure 2A, the proliferation rate of the glioma cells was reduced by 90% (± 5%) and 55% (± 10%) in the presence of the ribozyme or its mutant form respectively. A significant difference between DOTAP-, ribozyme- and mutant ribozyme-treated cells was found ($P < 0.0007$). The antiproliferative effect of the active ribozyme was significantly higher than its mutant form ($P < 0.0003$). Notably, the effect of the mutant ribozyme on cell proliferation is also significant ($P < 0.0026$). The inhibition effect of the mutant ribozyme is more likely to be due to its antisense activity.

To see whether the inhibition of cell proliferation after ribozyme treatment was reflected at the protein level, cell extracts obtained from control and test molecule-treated cells were subjected to Western blot analysis. In ribozyme-treated cells the amount of PKCα was reduced by approximately 73% (± 5%) ($P < 0.0001$) and this of Bcl-$x_L$ was reduced by 90% (± 15%) ($P < 0.0001$) as compared to controls. This result would suggest a possible interaction between PKCα and Bcl-$x_L$ proteins. A significant inhibition (50% ± 10%) of PKCα gene expression was also seen in mutant ribozyme-treated cells ($P < 0.002$). Ribozyme inhibition was isotype specific since the PKCδ levels were unaffected by any of the treatments (Figure 2B). The detection of the PKCα protein in the ribozyme-treated cells may not be surprising, since it has a long half-life in glioma cells (> 25 h). Analysis of the PKCα mRNA in DOTAP-, mutant ribozyme-, and ribozyme-treated cells by RT-PCR (Figure 2C) shows a dramatic reduction in PKCα signal in ribozyme-treated cells as compared to mutant ribozyme (PKC Rzm). This result would indicate that the inhibition effect of the ribozyme on PKCα gene expression is due to its cleavage activity of the mRNA as demonstrated previously (Sioud and Drlica, 1991).

### Effect of ribozyme-mediated loss of PKCα protein on induction of apoptosis

Morphological examination of glioma cell lines treated with the PKCα ribozymes indicated alteration in cellular morphology. Cells became rounded and displayed condensation of the nuclear chromatin as shown in Figure 3B. These morphological changes are reminiscent of apoptosis. The extent of apoptosis in the presence or absence of the ribozyme was assessed by the percentage of apoptotic nuclei visualized by propidium iodide staining (Figure 3C). In cells treated with ribozyme for 48 h, the percentage of

 *© 1999 Cancer Research Campaign*

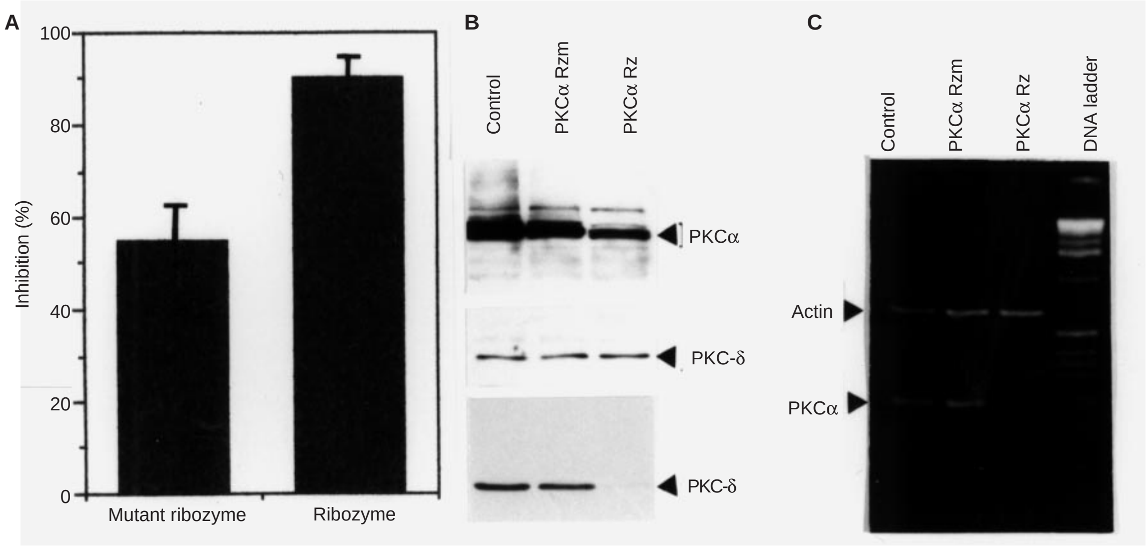

**Figure 2** Inhibition of glioma cell growth and PKCα gene expression by the ribozyme. (**A**) Inhibition of cell proliferation. Cells were transfected for 48 h and cell proliferation was measured by the MTT assay. Inhibition was expressed as a percentage of the DOTAP-treated cells. (**B**) The ribozyme reduced the expression of the PKCα and Bcl-$x_L$, but not PKCδ. After 48 h transfection time, protein extracts were prepared from DOTAP- (control), mutant ribozyme- and ribozyme-treated cells and 15 μg from each sample were analysed by Western blot using specific antibodies. (**C**) The ribozyme eliminated its target mRNA in the cell. Total RNA was prepared and the expression of PKCα was detected by RT-PCR as described in Materials and Methods. Data are representative of four independent experiments apoptotic nuclei was 87% as compared to only 5% in control cells. A significant fraction (30%) of mutant ribozyme-treated cells are apoptotic. That ribozyme-treated glioma cells were killed by apoptosis was confirmed by the use of the deoxynucleotidyl TUNEL assay. This method is based on the fact that apoptotic cells contain free 3′-end of double-stranded DNA due to the endonuclease digestion of genomic DNA at the nucleosomal intervals. Fluorescein isothiocyanate (FITC)-conjugated dUTP molecules were added to these 3′-end using the terminal deoxynucleotidyl transfererase enzyme (Figure 3D). As can be seen, all ribozyme-treated cells were in apoptotic stage. The induction of cell death in human glioma cells following ribozyme treatment was further confirmed by the presence of a DNA ladder which directly reflects the endonucleotic cleavage of chromosomal DNA typically associated with the apoptotic process (Figure 4, lane 3).

### DISCUSSION

This study demonstrated that inhibition of endogenous PKCα synthesis by a ribozyme induces apoptosis in cultured malignant gliomas, supporting an essential survival function for PKCα in these cells. The inhibition effect is specific, since the expression of the PKCδ isoform was not affected by the ribozyme treatment. Furthermore, the study indicates that the expression and/or the activity of the cell survival molecules Bcl-$x_L$ is under the control of PKCα signal pathway. This observation is important, because it links the PKCα isoform with apoptosis.

Prior studies have demonstrated that the PKC inhibitors are effective apoptotic-inducing agents, but do not identify the role played by each PKC isoform in this process. Further support for the involvement of PKCα in apoptosis is also provided by Whelan and Parker (1998) who found that loss of PKCα function correlated with the induction of apoptosis in COS cells. Furthermore, a recent study also showed that Bcl-2 phosphorylation by PKCα is required for its anti-apoptotic function. In fact, it was demonstrated that overexpression of PKCα in REH cells resulted in increased mitochondrial PKCα localization, augmented Bcl-2 phosphorylation and enhanced resistance to apoptosis induced by anticancer drugs (Ruvolo et al, 1998). A significant fraction of the PKCα was found to be associated with the mitochondria fraction prepared from glioma cells (data not shown). The biological significance of this mitochondrial PKCα are under investigation in our laboratory. In addition to its potential anti-apoptic function, PKCα was also found to be associated with multidrug resistance (MDR) and its inhibition by antisense attenuated the MDR phenotype (Blobe et al 1994; Caponigro et al, 1997).

In addition to displaying a high PKC activity, glioma cells also overexpress the Bcl-$x_L$ protein that participate in the control of apoptosis (Sioud and Sørensen, 1998). As shown, PKCα ribozyme induced apoptosis concomitant with dramatic decreases in Bcl-$x_L$ protein. Bcl-2 gene expression was found to be inhibited by calphostin C (Ikemoto et al, 1995). Taken together these observations suggest that the expression and/or the activity of Bcl-2 and Bcl-$x_L$ are modulated by PKC, in particular the PKCα. Increased expression of Bcl-$x_L$ protein in glioma cells may contribute to their drug resistance, since its overexpression in murine FL5.12 cells conferred a MDR phenotype (Min et al, 1995). Based upon the data presented here, it appears that PKCα targeting in glioma cells is more likely to be an appropriate target, since its inhibition by the ribozyme simultaneously inhibited the expression of Bcl-$x_L$ resulting in cell death by apoptosis. In addition to Bcl-$x_L$ down-regulation, other genes could be affected by the down-regulation of PKCα. In this respect, analysis of control and ribozyme-treated cells by the PCR-based differential display technique identify some differentially expressed cDNA bands which are under investigation in our laboratory.

Our present study and early reports using PKC inhibitors and antisense deoxynucleotides underlie the importance of PKC, in particular PKCα, in sustained tumour growth. Among the anti-PKC drugs, bryostatin and staurosporine derivatives such as UCN-01 and CGP41251 have been evaluated in patients with cancer (Caponigro et al, 1997). The present study would support

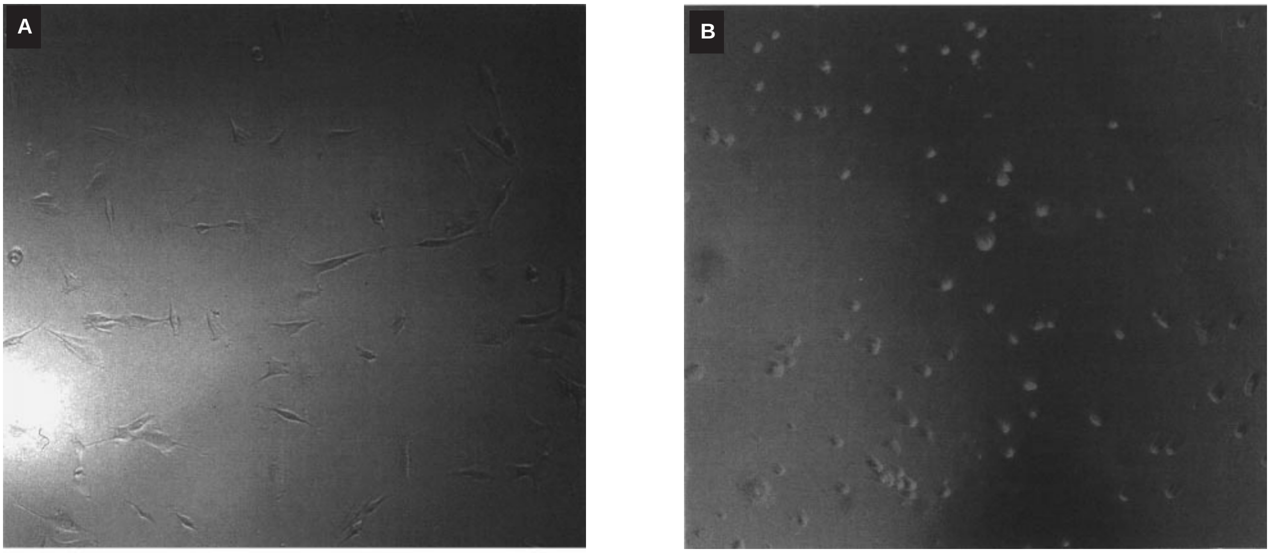

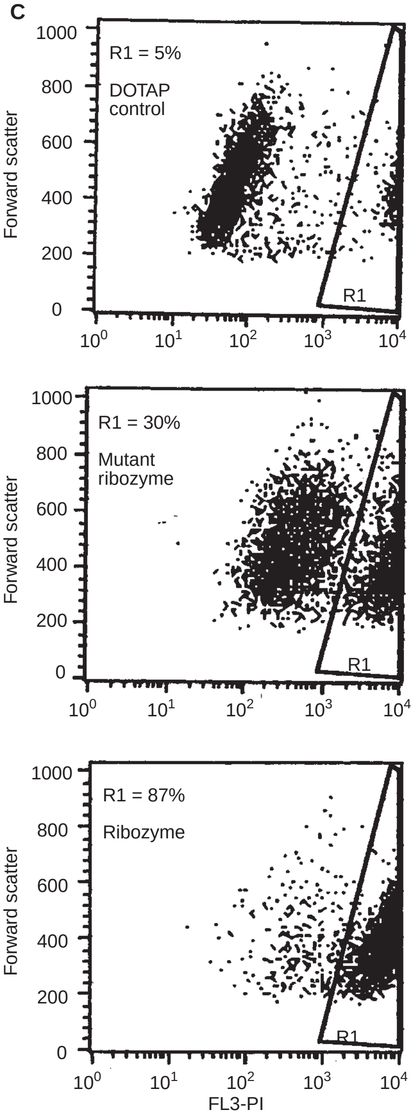

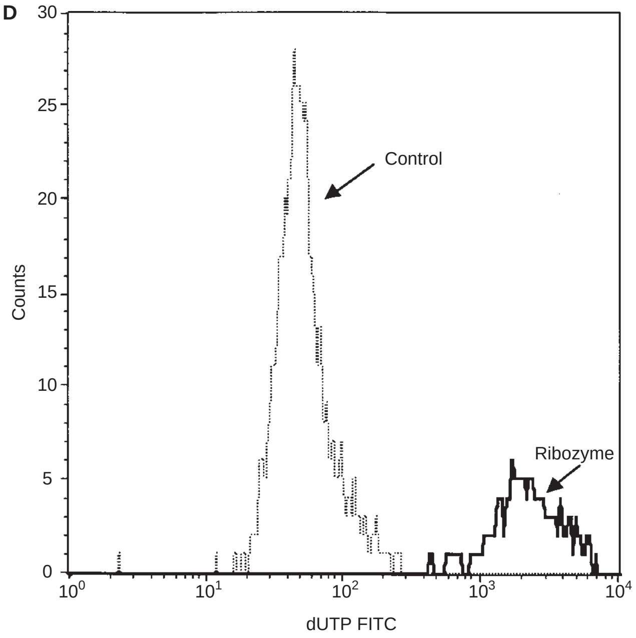

**Figure 3** Ribozyme inhibition of the PKCα gene expression induces apoptosis in glioma cells. Light microscope image of PKCα ribozyme treated U87MG cells. DOTAP- (**A**) and ribozyme-treated cells (**B**) for 48 h were photographed to illustrate the morphological changes produced by the inhibition of the PKCα isoform. (**C**) Cell death in glioma cells as detected by propidium iodide (PI) positive cells. DOTAP-, mutant ribozyme- and ribozyme-treated cells for 24 h were stained with PI and analysed by flow cytometry. (**D**) Quantification of cellular DNA fragmentation by the TUNEL method. DOTAP- and ribozyme-treated cells for 48 h were analysed by the TUNEL method as described in Materials and Methods. Data are representative of four independent experiments.

 *© 1999 Cancer Research Campaign*

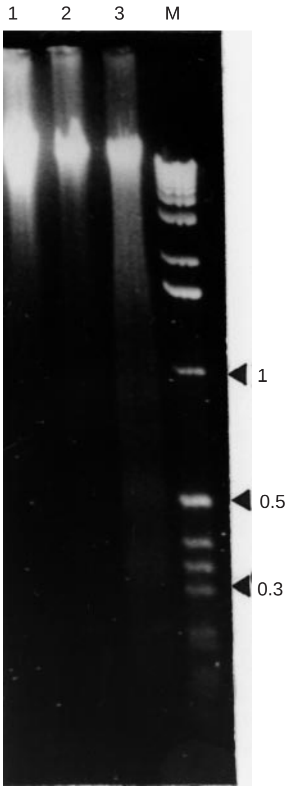

**Figure 4** Induced DNA fragmentation by the ribozyme. Following 24-h transfection time, cells were lysed, DNA crude preparations were prepared, analysed by a 1% agarose gel and then stained with ethidium bromide. Lane 1, DOTAP-treated cells; lane 2, mutant ribozyme-treated cells; lane 3, ribozyme-treated cells. M, 1 Kb DNA ladder. A 'DNA ladder' is evident in ribozyme-treated cells. Data are representative of four independent experiments these clinical trials and strengthens the rationale to plan phase 2 clinical trials with these agents, including PKCα ribozyme and antisense in patients with gliomas. For this to be feasible, work should be conducted to investigate the potential of these drugs to cross the brain barrier. However, free ribozymes or capsules containing ribozymes can be injected into the tumour. This strategy may offer many advantages over systemic therapy, since it would ensure a high concentration of the drug within the tumour and more importantly would be less toxic to normal cells.

In conclusion, we have demonstrated that a selective inhibition of PKCα gene expression by a ribozyme decreases proliferation of human glioma cell lines in vitro by activating the apoptotic process. Thus, our data demonstrate for the first time that the machinery of apoptosis in cancer cells can be targeted specifically by ribozymes and suggest a potential interaction between PKCα and the Bcl-$x_L$ protein.

### ACKNOWLEDGEMENTS

This work was supported by the Norwegian Radium Hospital and the Norwegian Research Council.

### REFERENCES

Blobe GC, Obeid LM and Hannun YA (1994) Regulation of protein kinase C and role in cancer biology. *Cancer Met Rev* **13**: 411–431

Cameron FH and Jennings PA (1989) Specific gene suppression by engineered ribozymes in monkey cells. *Proc Natl Acad Sci USA* **83**: 8859–8862

Caponigro F, French RC and Kaye SB (1997) Protein kinase C: a worthwhile target for anticancer drugs? *Anti-Cancer Drugs* **8**: 26–33

Chomczynski P and Sacchi N (1987) Single step method of RNA isolation by acid guanidinium thiocyanate phenol chloroform extraction. *Anal Biochem* **162**: 156–159

Cotten M and Birnstiel ML (1989) Ribozyme mediated destruction of RNA in vivo. *EMBO J* **8**: 3861–3866

Couldwell WT, Hinton DR, He S, Chen TC, Sebat I, Weiss MH and Law RE (1994) Protein kinase C inhibitors induce apoptosis in human malignant glioma cell lines. *FEBS Lett* **345**: 43–46

Couldwell WT, Uhm JH, Antel JP and Young VW (1991) Enhanced protein kinase C activity with the growth rate of malignant gliomas in vitro. *Neurosurgery* **29**: 880–887

Dean N, McKay R, Miraglia L, Howard R, Cooper S, Giddings J, Nicklin P, Meister L, Ziel R, Geiger T, Muller M and Fabbro D (1996) Inhibition of growth of human tumor cell lines in nude mice by an antisense oligonucleotide inhibitor of protein kinase C-α expression. *Cancer Res* **56**: 3499–3507

Dekker LV and Parker PJ (1994) Protein kinase C: a question of specificity. *TIBS* **19**: 73–77

Haseloff J and Gerlach WL (1988) Simple RNA enzymes with new and highly specific endoribonuclease activities. *Nature* **334**: 585–591

Hertel KJ, Pardi A, Uhlenbeck OL, Koizumi M, Ohtsuka E, Vesugi S, Cedergren R, Eckstein F and Gerlach WL (1992) Numbering system for the hammerhead. *Nucleic Acids Res* **20**: 3252

Ikemoto H, Tani E, Matsumoto T, Nakano A and Furuyama JI (1995) Apoptosis of human glioma cells in response to calphostin C, a specific protein kinase C inhibitor. *J Neurosurg* **83**: 1008–1016

Kroemer G (1997) The prot-oncogene Bcl-2 and its role in regulating apoptosis. *Nat Med* **3**: 614–620

Meggio F, Donnella-Deana A, Ruzzene M, Brunati AM, Cesaro L, Buerra B, Meyer T, Mett H, Fabbro D, Furet P, Dobrowolksa G and Pinna LA (1995) Different susceptibility of protein kinases to staurosporine inhibition: kinetic studies and molecular bases for the resistance of protein kinase CK2. *Eur J Biochem* **234**: 317–322

Min AJ, Rudin CM, Boise LH and Thompson CB (1995) Expression of Bcl-$x_L$ can confer a multidrug resistance phenotype. *Blood* **86**: 1903–1910

Mischak H, Goodnight J, Kolch W, Martiny-Baron G, Schaechtle C, Kazanietz MG, Blumberg PM, Pierce JH and Mushinski JF (1993) Overexpression of protein kinase C-δ and -ε in NIH 3T3 cells induces opposite effects on growth, morphology, anchorage dependence, and tumorgenicity. *J Biol Chem* **268**: 6090–6096

Newton AC (1995) Protein kinase C: structure, function, and regulation. *J Biol Chem* **270**: 28495–28498

Nishizuka Y (1992) Intracellular signaling by hydrolysis of phospholipids and activation of protein kinase C. *Science* **258**: 607–614

Parker P, Kour G, Marais R, Mitchell F, Pears C, Stabel S and Webster C (1989) Protein kinase C a family affair. *Mol Cell Endocrinol* **65**: 1–11

Pegoraro L, Palumbo A, Erikson J, Falda M, Giovanazzo B, Emanuel BS, Rovero G, Nowell PC and Croce CM (1984) A 14;18 and a 8;14 chromosome translocation in a cell line derived from an acute B cell leukemia. *Proc Natl Acad Sci USA* **81**: 7166–7170

Qiao L, Koutsos M, Tsai LL, Kozoni V, Guzman J, Shiff SJ and Rigas B (1996) Staurosporine inhibits the proliferation, alters the cell cycle distribution and induces apoptosis in HT-29 human colon adenocarcinoma cells. *Cancer Lett* **107**: 83–89

Rodin CM and Thompson CB (1997) Apoptosis and disease. *Annu Rev Med* **48**: 267–281

Ruvolo PP, Deng X, Carr BK and May WS (1998) A functional role for mitochondrial protein kinase Cα in Bcl-2 phosphorylation and suppression of apoptosis. *J Biol Chem* **273**: 25436–25442

Sarver N, Cantin EM, Chang PS, Zaia JA, Ladne PA, Stephens DA and Rossi JJ (1990) Ribozymes as potential anti HIV-1 therapeutic agents. *Science* **247**: 1222–1225

Scanlon KJ, Ishida H and Kashani-Sabet M (1994) Ribozyme-mediated reversal of the multidrug-resistant phenotype. *Proc Natl Acad Sci USA* **91**: 11123–11127

Sioud M (1994) Interaction between tumor necrosis factor α ribozyme and cellular proteins. *J Mol Biol* **242**: 619–629

Sioud M (1996) Ribozyme modulation of lipopolysaccharide-induced tumor necrosis factor-α production by peritoneal cells in vitro and in vivo. *Eur J Immunol* **26**: 1026–1031

Sioud M and Drlica K (1991) Prevention of HIV-1 integrase expression in *E coli* by a ribozyme. *Proc Natl Acad Sci USA* **88**: 7303–7307

Sioud M and Jespersen L (1996) Enhancement of hammerhead ribozyme catalysis by glyceraldehyde-3-phosphate dehydrogenase. *J Mol Biol* **257**: 775–789

Sioud M and Sørensen DR (1998) A nuclease-resistant protein kinase Cα ribozyme blocks glioma cell growth. *Nature Biotech* **16**: 556–561

Sioud M, Natvig JB and Førre Ø (1992) Preformed ribozyme destroys tumour necrosis factor mRNA in human cells. *J Mol Biol* **223**: 831–835

Whelan RDH and Parker PJ (1998) Loss of protein kinase C function induces an apoptotic response. *Oncogene* **16**: 1939–1944

 *© 1999 Cancer Research Campaign*
